# Ongoing epidemic of lymphogranuloma venereum in HIV-positive men who have sex with men: how symptoms should guide treatment

**DOI:** 10.7448/IAS.17.4.19657

**Published:** 2014-11-02

**Authors:** Gerrit Mohrmann, Christian Noah, Michael Sabranski, Hany Sahly, Hans-Jürgen Stellbrink

**Affiliations:** 1 Infektionsmedizin, Labor Lademannbogen MVZ GmbH, Hamburg, Germany; 2 Study Center, Infektionsmedizinisches Centrum Hamburg, Hamburg, Germany

## Abstract

**Introduction:**

Lymphogranuloma venereum (LGV) is a sexually transmitted infection (STI) caused by chlamydia trachomatis (CT) genotype L (L1, L2 and L3). Recent outbreaks of LGV in Europe and North America affected mainly men who have sex with men (MSM). Infections with CT serotypes D-K are mostly associated with mild symptoms or may be clinically silent. However, infections with L genotypes are more invasive and induce anogenital ulcer or inguinal lymphadenopathy.

**Materials and Methods:**

Between 2003 and 2013, anal or genital CT infections were diagnosed in 471 symptomatic patients attending the Infectious Diseases Center Hamburg (ICH). CT DNA was detected by PCR. CT genotyping of positive samples was performed by sequence analyses of omp1 PCR products (samples between 2003 and 2010). Samples between 2012 and 2013 were analyzed by genotype L specific real-time PCR.

**Results:**

In total, 221 LGV patients were identified (3 in 2003; 11 in 2004; 26 in 2005; 33 in 2006; 24 in 2007; 16 in 2008; 18 in 2009; 15 in 2010; 17 in 2011; 26 in 2012 and 32 in 2013). One hundred ninety-eight of 221 (89.6%) patients with LGV were HIV-infected; 10 of 221 (4.5%) were HIV-negative, and the HIV-status unknown in 13 (5.9%). The majority of LGV positive patients (204/221; 92%) had anorectal symptoms (bloody proctitis, rectal pain, purulent or mucous discharge, tenesmus, constipation), compared to 17/221 (8%) who presented with genital symptoms as urethritis, genital ulcer or inguinal lymphadenopathy. Of 283 CT-positive patients with anorectal symptoms, genotype L was detected in 205 (72%), while non-L genotypes (D-K) were found in 78 (28%). In contrast, genotype L was found in only 6% of patients with genital symptoms (11/177), whereas 94% (166/177) were infected with non-L genotypes only.

**Conclusions:**

The epidemic of LGV among predominantly HIV+ MSM is ongoing. LGV might be underdiagnosed, especially in patients with anorectal symptoms. Infections with CT serotypes D-K are more often associated with urogenital symptoms or asymptomatic infection, whereas LGV genotypes are found most frequently in patients with rectal/intestinal symptoms. Anorectal CT infections in MSM should be further characterized by genotyping for LGV, as for LGV three weeks of doxycycline treatment are recommended. CT genotypes D-K generally require shorter antibiotic treatment. If CT genotyping is not available, the duration of treatment should be prolonged to three weeks empirically for CT-positive patients with anorectal or intestinal symptoms.

